# Comprehensive study on structural, electronic, optical, elastic, and transport properties of natural mercury sulphohalides via DFT computation

**DOI:** 10.1038/s41598-024-69430-3

**Published:** 2024-08-10

**Authors:** M. Hariharan, R. D. Eithiraj

**Affiliations:** Department of Physics, School of Advanced Sciences, Vellore Institute of Technology (VIT), Chennai, Tamil Nadu 600127 India

**Keywords:** Mercury sulphohalides, DFT, Indirect band gap, Elastic anisotropy, Lattice thermal conductivity, ZT, Condensed-matter physics, Theory and computation

## Abstract

The Mercury Sulphohalides have attracted significant attention in the fields of solar cells and thermoelectric applications. This study delves into the fundamental characteristics, including structural, elasticity, electronic behavior, phonon stability, optical properties, and transport features of AgHgSZ (Z = Br, I) through computational simulations based on Density Functional Theory (DFT) using WIEN2k software. Meticulous calculations of the phonon band structure ensure dynamic stability. The semiconductor nature with indirect band gaps (1.833 eV and 1.832 eV) for Mercury Sulphohalides (Br, I), as revealed by their band structures, suggests diverse photovoltaic and transport applications. Mechanical assessments show stable ductility for AgHgSBr and brittleness for AgHgSI, along with anisotropy and resistance to scratching. Optical properties exhibit anisotropy and significant UV absorption. Analysis of effective masses, exciton binding energy, and exciton Bohr radius suggests low exciton binding energy and classification under Mott-Wannier excitons. Positive thermopower results indicate holes as the predominant charge carriers in AgHgSBr and AgHgSI materials. Moreover, essential thermoelectric factors are examined, revealing the compounds’ potential for thermoelectric applications. Notably, the figure of merit (ZT) at 300 K for AgHgSBr and AgHgSI are calculated to be 0.41 and 0.13, respectively. While these values are low at 300 K, they indicate promising potential for thermoelectric applications at higher temperatures. In summary, this investigation provides valuable understanding into the photovoltaic and thermoelectric properties of AgHgSZ (Z = Br, I) materials, potentially paving the way for further exploration in this domain.

## Introduction

Natural mercury sulphohalides constitute a distinct group of minerals characterized by their specific geochemistry and crystal structure. Typically found in the oxidized zones of ore deposits containing primary mercury-bearing minerals like cinnabar or fahlore enriched with mercury, these minerals have the capacity to concentrate other elements such as bromine and iodine, which are often sparsely distributed in nature. This mineral family comprises ten distinct species, further categorized into two subgroups. The first subgroup consists around six minerals that have a general formula of Hg_3_S_2_Hal_2_ (where Hal represents halogen elements such as chlorine, bromine, or iodine), including three polymorphs of Hg_3_S_2_Cl_2_ (known as corderoite^1^, lavrentievite^2,3^, and kenhsuite^4^), along with dimorphous arzakite^2,3^ and grechishchevite^5–7^ via the ideal composition of Hg_3_S_2_Br_2_, and radtkeite with the formula Hg_3_S_2_ICl^8–10^. The second group is made up of four sulphohalide minerals that include both mercury and silver as metal cations: dimorphs of AgHgSCl (capgaronnite^11^ and iltisite^12^), perroudite with the composition Ag_4_Hg_5_S_5_(I,Br)_2_Cl_2_^13–15^, and the newly found hanauerite with the formula AgHgSI^16^. The crystal structure of hanauerite closely resembles that of its synthetic counterpart AgHgSI, as well as similar compounds such as CuHgSeBr and AgHgSBr^17^. Additionally, high-temperature phases like CuHgSCl^18^ and CuHgSBr^19^ share similar orthorhombic crystal structures in space group Pmma, with unit cell dimensions close to those of hanauerite.

Numerous experiments have been conducted on “coinage metal mercury chalcogenide halides”^20^. Beck et al. investigated the crystal structures of CuHgSBr^20^, CuHgSCl^20^, CuHgSeBr, AgHgSBr, and AgHgSI^17^ using single crystal specimens synthesised hydrothermally in hydrohalic acids at distinctive temperatures. Additionally, we already conducted first principles calculations on CuHgSBr and CuHgSeBr^21,22^ with a focus on their thermoelectric potential. However, theoretical calculations are not available for AgHgSZ (Z = Br, I); only experimental techniques are available^17^. Therefore, we conducted first principles calculations on the compounds AgHgSBr and AgHgSI (Mercury Sulphohalides) for thermoelectric applications.

In different energy utilization scenarios such as fossil fuel combustion, electricity generation, and solar energy harvesting, the production of waste heat is an unavoidable byproduct^23^. Frequently disregarded, this excess heat can pose risks, including the potential for damage to computational and data storage systems due to heat buildup. Thermoelectric (TE) devices provide a solution by converting a portion of this waste heat directly into electrical energy to operate the system, while the remaining electrical energy is used at a specific level to maintain system stability. It can be used to cool areas^24–27^. Recent research reveals that the ZT value of n-type Mg_3_Bi_2_-based materials is about ~ 0.9 at 350 K, and TE coolers can generate temperature differences of about ~ 91 K^28^, which is in contrast to previous data. This is higher than the reported 81 K^29^. With no moving parts, noise, or greenhouse gas emissions, solid-state thermoelectric (TE) devices are ideal for miniaturization. The effectiveness of TE devices is dependent on the performance of TE materials, which enable effective heat conversion into electricity and refrigeration, as indicated by high figure-of-merit (ZT) values. At the moment, most TE devices and materials have energy conversion efficiencies of less than 10% and ZT values of about 1.0^30^. However, achieving a ZT value of four could potentially lead to an energy conversion efficiency of 30%, presenting an enticing prospect for practical systems^31–33^. Furthermore, obtaining a ZT value of three may make thermoelectric (TE) household refrigerators more economically competitive than traditional compressor-based ones. Slack was the first to introduce the concept of a “phonon glass electron crystal” (PGEC), referring to a material with ideal ZT features including high mobility charge carriers and glass-like thermal conductivity. In contrast, fine-tuning specific characteristics in bulk materials to achieve this goal is a difficult process^34^. In 2012, researchers expanded on the PGEC concept by introducing a fresh technique called as “phonon-liquid electron-crystal” (PLEC). This method leverages superionic conductors' liquid-like behaviour to reduce thermal conductivity (κ_L_) to glass levels^35^.

For instance, many of the materials with exceptionally high ZT values considered for power generation, such as Bi_2_Te_3_^29,36^, PbTe^37,38^, and GeTe^39,40^, rely on tellurium, a rare and consequently expensive element. SnSe demonstrates impressive ZT values attributed to its exceptionally low thermal conductivity and advantageous electronic characteristics upon doping^41^. However, its high-temperature stability is constrained^42^. Although Half-Heusler materials demonstrate substantial power factors (S^2^σ), their overall ZT remains constrained by their inherently high thermal conductivity^43,44^. In the present study, we performed Density Fuctional Theory (DFT) computation on Mercury Sulphohalides AgHgSZ (Z = Br, I) to estimate the various physical properties.

## Method of calculations

In this current investigation, computations were carried out on Mercury Sulphohalides represented by the chemical formula AgHgSZ (Z = Br, I) using the WIEN2k code^45,46^ (Version: 18.2, http://susi.theochem.tuwien.ac.at/). Using Murnaghan's equations, the PBE-GGA method was carefully used to compute a number of parameters, including the bulk modulus, ground state characteristics, and lattice constant. Although this approximation method is effective in determining structural parameters, it has limitations in accurately estimating electronic properties, particularly the band gap. To do exact band gap computations, the TB-mBJ functional was used, which takes less computing time than the HSE06 function^47^. For inorganic materials, the outcomes of the TB-mBJ and HSE06 functionals are essentially the same. As for organic-based materials with doping compositions that fall below 0.25, the HSE06 potential accurately represents them, whereas the TB-mBJ functional tends to underestimate the band gap values. Despite the fact that the HSE06 potential requires substantial computational resources and time, the TB-mBJ functional is significantly less computationally expensive and time-consuming. In the case of simple inorganic materials, we propose using TB-mBJ because it provides a good mix of reliability and computational capacity^48^.

Hence, the TB-mBJ functional is utilized in conjuction with PBE-GGA for precise band gap calculations. All ground state properties discussed in this paper are determined using PBE-GGA, based on the volume optimization and total energy. Furthermore, the table includes the reported band gap values obtained with TB-mBJ. For precise computations, essential parameters including “L_max_ = 10, G_max_ = 12, and R_MT_ x K_max_ = 8” are specified as inputs in the WIEN2k package. The muffin tin radii values for Silver (Ag), Mercury (Hg), Sulfur (S), Bromine (Br), and Iodine (I) are 2.5, 2.5, 1.95, 2.45, and 2.5 atomic units (a.u.), respectively. Additonally, a K-point of 100 has been employed to achieve converged parameters, with a merging threshold set at 10^–4^ Ry. The optical properties were calculated using OPTIC, as implemented in WIEN2k. The effective masses of electrons and holes were estimated from the band structure diagram. Thermoelectric properties are assessed using BoltzTrap code, whereas the phonon thermal conductivity (κ_L_) is elucidated manually by utilizing the “Slack equation”^49^.

## Results and discussion

### Crystal structure and structural optimization

**Figure 1:**
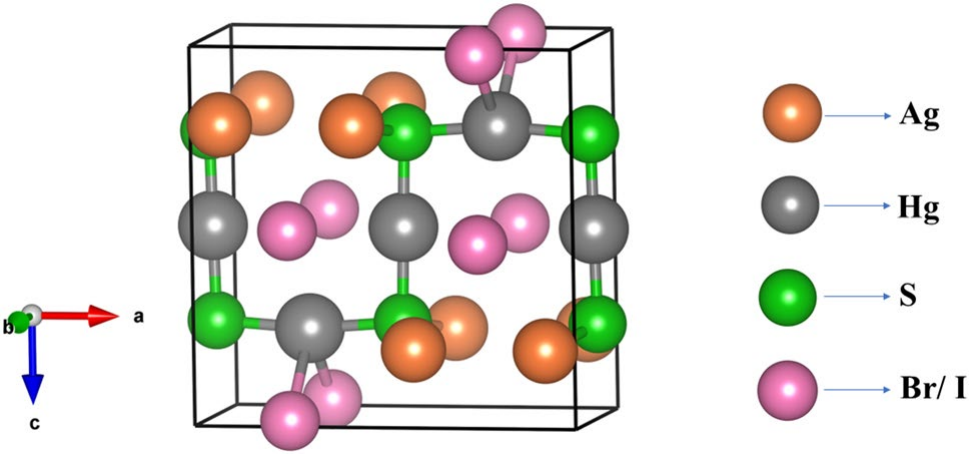
Crystal structure of Mercury Sulphohalides AgHgSZ (Z = Br, I).

**Figure 2:**
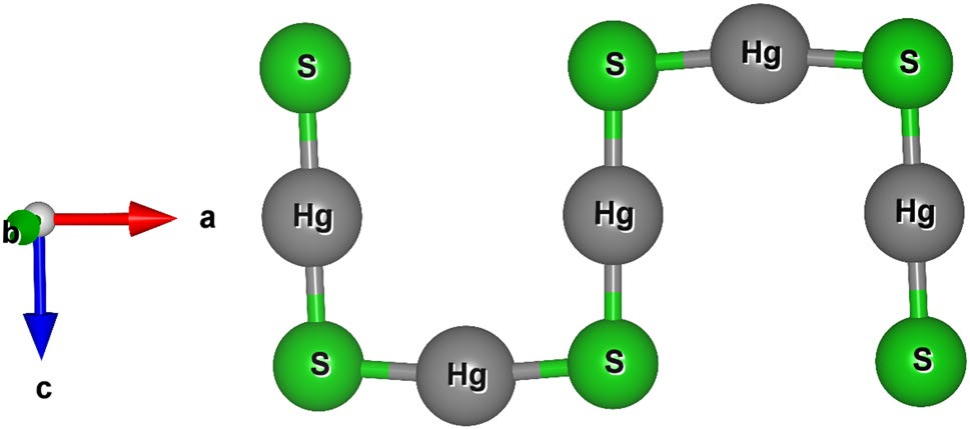
Unit cell of Hg-S chains for Mercury Sulphohalides AgHgSZ (Z = Br, I).

AgHgSZ (Z = Br, I) exhibit iso-typical crystallization. The unit cell structure and crystallographic arrangement of AgHgSBr and AgHgSI reveals a planar arrangement comprising Hg and S atoms forming chains, accompanied by markedly distorted planar M_2_X_2_ rings, depicted in Figs. 1 and 2. The mercury atoms, denoted as Hg(1) and Hg(2), along with the halogen atoms, occupy specific twofold lattice positions characterized by the symmetry mm2 or 2/m. Conversely, atoms of type M (where M = Cu, Ag) and the chalcogen atoms occupy distinct fourfold lattice positions under the symmetry m. Each elementary unit consists of four formula units. These structures consist of two primary units: a flat chain made up of Hg and S atoms, displaying considerable distortion. The construction of these HgY chains involves two distinct crystallographic configurations of mercury atoms, Hg(1) and Hg(2), along with a chalcogen atom S. Hg(1) and Hg(2) have strikingly comparable coordination environments, with both being essentially linearly coordinated by two chalcogen atoms S. The Hg–S distances in the sulfur-containing compounds range from 2.36 (Å) to 2.38 (Å) are represented in the Table 1, consistent with experimental observations in cinnabar (Hg–S: 2.36 (Å)^17^).

**Table 1:** The computed bond length in the crystal structure for AgHgSBr and AgHgSI compounds.

Material	Bond	Bond distance (Å) (Theoretical)	Bond distance (Å) (Experimental^17^)
AgHgSBr	Ag–Br	3.1095	3.1090
	Hg–S	2.3626	2.3540
	Ag–S	2.4980	2.4980
	Hg–Hg	3.4957	–
AgHgSI	Ag–I	3.2093	3.2090
	Hg–S	2.3758	2.3760
	Ag–S	2.5349	2.5350
	Hg–Hg	3.5975	–

**Figure 3:**
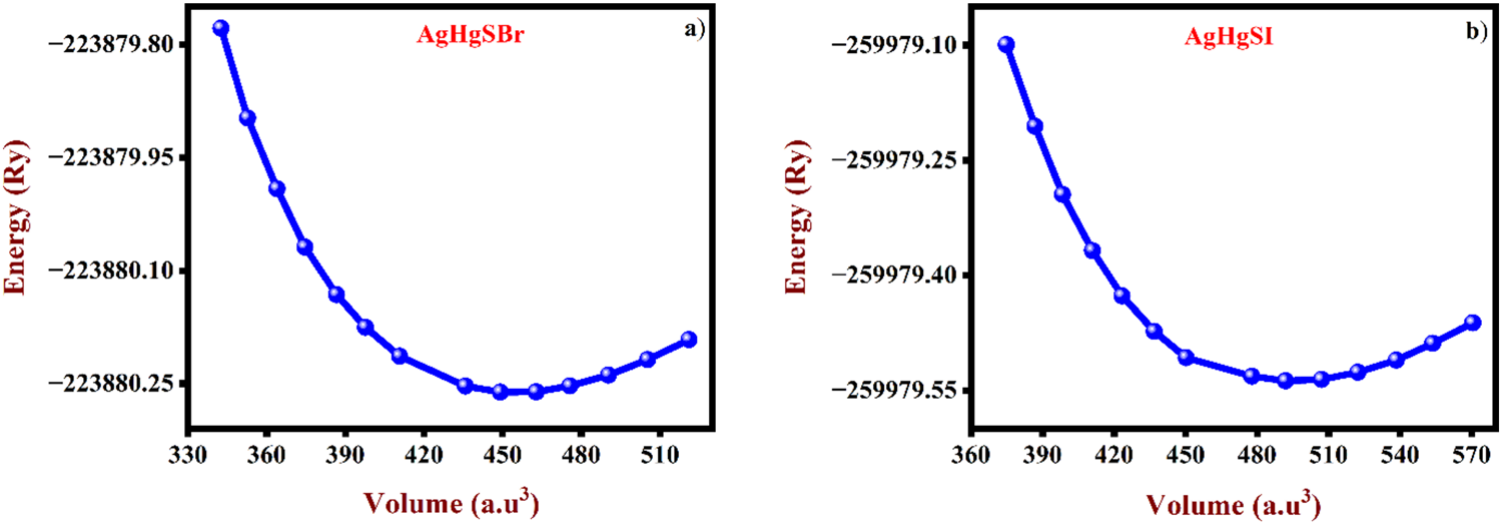
Geometry optimization (volume vs energy) for (**a**) AgHgSBr and (**b**) AgHgSI.

As seen in Fig. 3a,b, we evaluated energy vs volume and used Murnaghan's equation of states to analyse the results in order to find the lattice parameter for AgHgSBr and AgHgSI as well as the best configuration for the unit cell. As compared to the TB-mBJ functional, the equilibrium volume predicted using GGA-PBE closely matches experimental results, demonstrating the superiority of the former. Consequently, we determine all physical characteristics using the GGA approximation. The bandgap and total energy computed using mBJ and GGA-PBE functionals are summarised in Table 2. Furthermore, Table 3 shows the volume variation, equilibrium volume and optimised lattice constants of the GGA-PBE functional, which demonstrates strong agreement with the experimental work.

**Table 2:** Computed band gaps and total energy of AgHgSBr and AgHgSI compounds using different functionals.

Material	XC-Functionals	E_g_ (eV)	E_0_ (Ry)
AgHgSBr	GGA-PBE	1.833	−223,880.236
	mBJ	2.717	−223,716.697
AgHgSI	GGA-PBE	1.832	−259,979.517
	mBJ	2.660	−259,796.289

**Table 3:** Elucidated the optimized lattice constants, unit cell volume and volume variation for AgHgSBr and AgHgSI compounds using different functionals.

Material (GGA)	a (Å)	b (Å)	c (Å)	V (Å^3^)	Volume variation, (%)
AgHgSBr Experimental^17^	9.648	4.661	9.426	423.88	−5.766
Theoretical	9.841	4.754	9.615	449.82	
AgHgSI Experimental^17^	10.159	4.647	9.849	464.99	−5.721
Theoretical	10.361	4.739	10.045	493.21	

### Electronic properties

**Figure 4:**
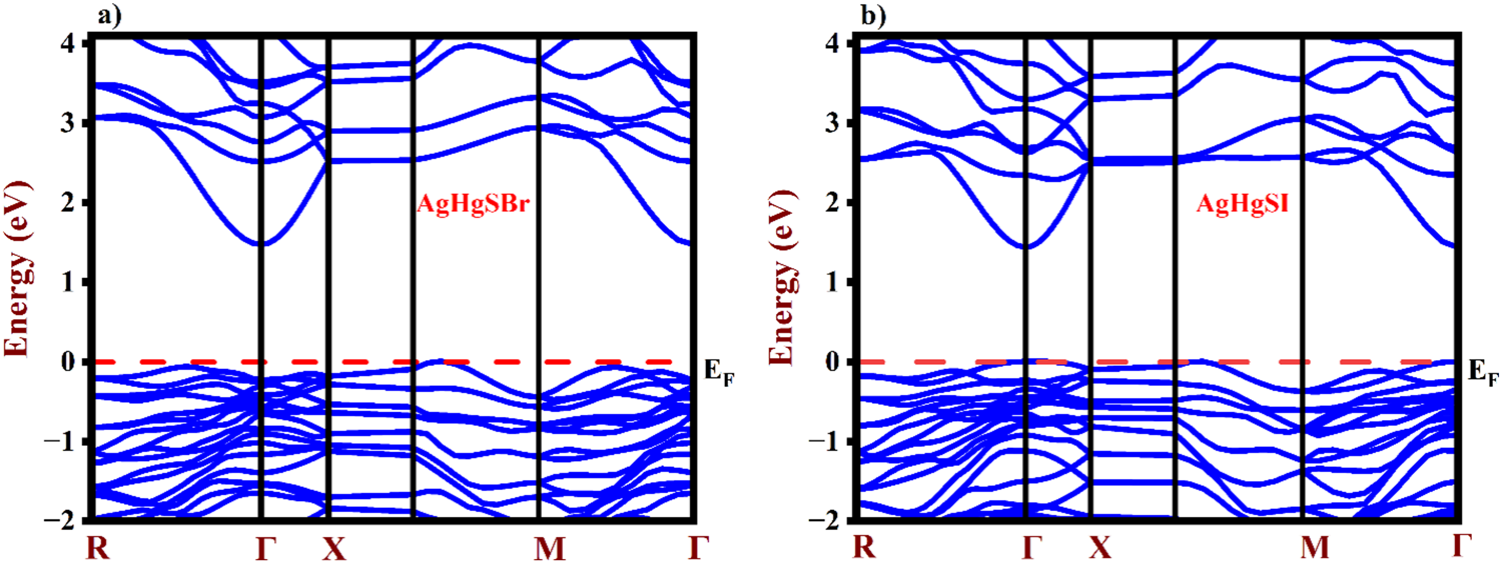
Energy band structure of (**a**) AgHgSBr and (**b**) AgHgSI.

Semiconductors, insulators, metals, and semimetals can each be distinguished by their unique electronic properties, which play a crucial role in understanding carrier transport mechanisms. Consequently, electronic band structure calculations can provide valuable insights into the suitability of materials for specific commercial applications. The band structures (BS) of AgHgSZ (Z = Br, I) were analyzed using both the TB-mBJ and PBE-GGA functionals. According to Table 2, the computed indirect band gaps (BS), which are shown in Fig. 4a,b, for AgHgSBr and AgHgSI using PBE-GGA are 1.833 eV and 1.832 eV, respectively. In AgHgSBr, the valence band lies within the asymmetry point, bridging the M and X directions, while conduction band occupies the Γ direction. For AgHgSI, valence band extends between the Γ and X directions, whereas the conduction band remains in the Γ direction. This arrangement indicates an indirect transition that benefits thermoelectric qualities but may have a little impact on optical properties. The bandgaps investigated make these materials promising candidates for solar cells and photovoltaic applications. AgHgSI has a lower band gap than AgHgSBr due to iodine's (I) higher ionic radius. This is explained by a downward shift of the atomic orbitals and a decrease in energy levels inside the conduction band. Moreover, the valence band (VB) shows a relatively flat distribution with fewer states and suggests that holes are the dominant carriers, whereas the conduction band (CB) has a tighter packing of states. This discovery also implies that holes have greater effective masses than electrons, which impacts thermoelectric properties.

The optical performance of the device is significantly influenced by electron transitions between VB and CB, which are the basis for understanding optoelectronic attributes. Additionally, Fig.s 5a,b, 6a,b demonstrate the density of states for AgHgSBr, AgHgSI, Ag, Hg, S, and Br/I. The CB is dominated by Br d states with energies ranging from 5 to 17.31 eV for AgHgSBr and 2.42–17.31 eV for AgHgSI. The VB band is predominantly made up of Ag *d* states with energies ranging from 0 to − 5.0 eV, as well as Br *p* and Hg *d* states. Consequently, our analysis demonstrates considerable electron transfers from Ag *d* states to Br *d* states, altering the optical characteristics of solar cells and other optical consequences through the recombination process. Furthermore, we removed certain orbitals due to their low energy.

**Figure 5:**
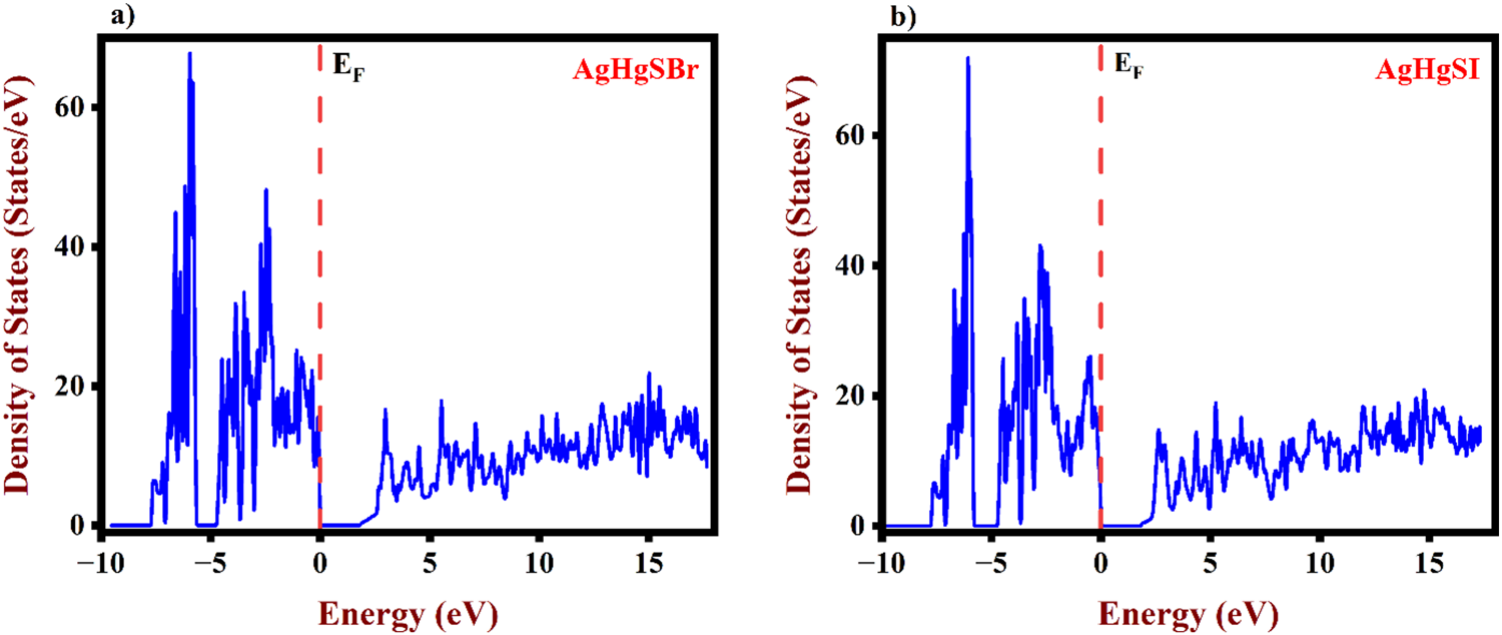
TDOS of (**a**) AgHgSBr and (**b**) AgHgSI using GGA-PBE.

**Figure 6:**
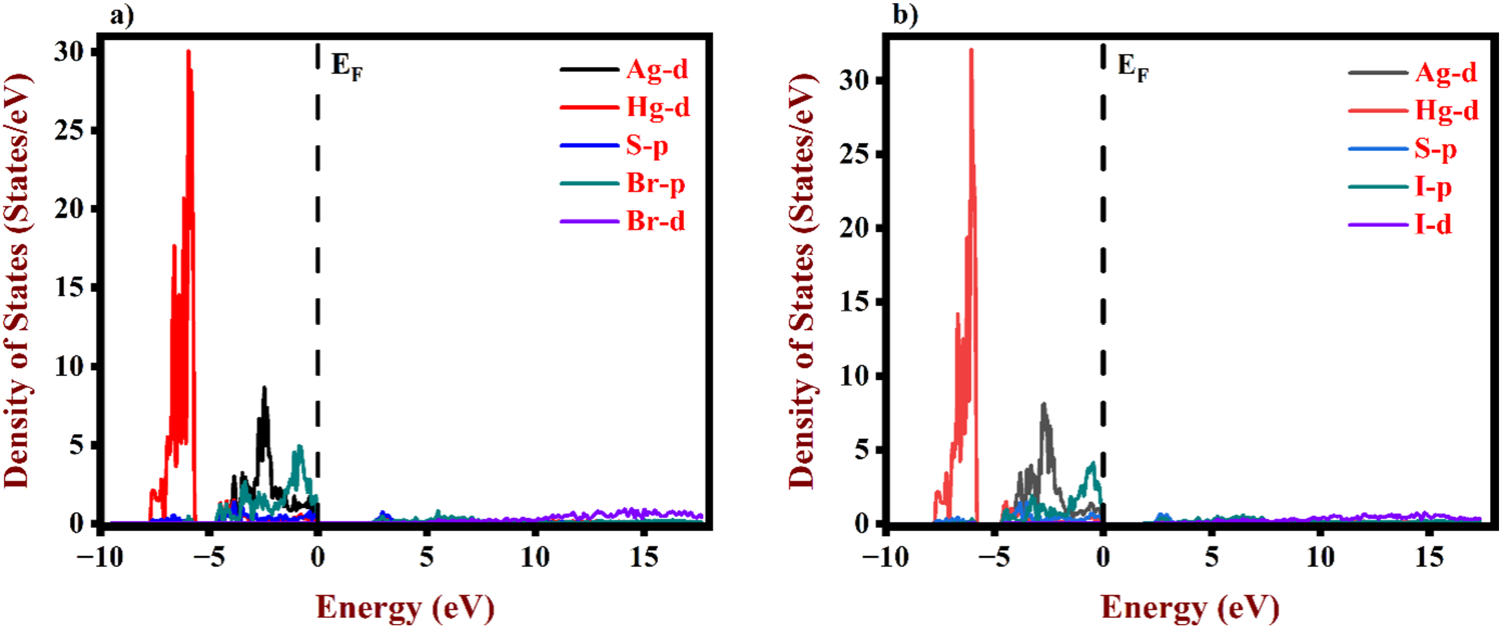
PDOS of (**a**) AgHgSBr and (**b**) AgHgSI using GGA-PBE.

### Dynamical stability

**Figure 7:**
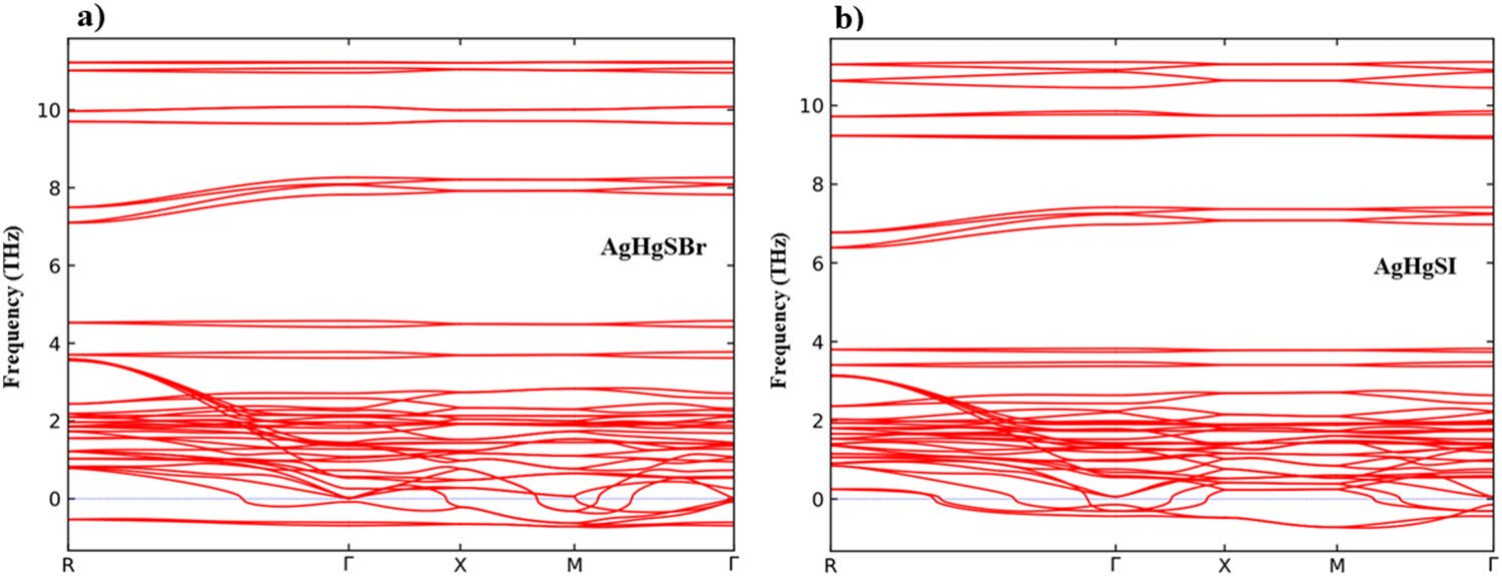
Phonon band structure (**a**) AgHgSBr and (**b**) AgHgSI.

To confirm the dynamic stability, we carried phonon band structure computations with the PHONOPY algorithm^50^ combined with the WIEN2k code. The dimensions of the supercell were set as “1 1 1”, while the q-point mesh was specified as 11 × 11 × 11, resulting in 1331 points. Figure 7a,b present the phonon dispersion spectra for AgHgSBr and AgHgSI, showcasing their distinctive behaviors along the k-points direction R-Γ-X-M-Γ. The presence of imaginary (negative) frequency components within these spectra indicates dynamic instability, hinting at the potential for a structural phase transition. As a result, the current configuration is regarded as metastable. The Ag, Br, and Hg atoms are thought to be the origin of this instability as they produce negative frequencies and a significant imaginary mode amplitude. Moving forward, adjustments to the lattice parameter are required, along with a thorough examination of the phonon band structure to assess structural stability. Future investigations could explore the nature of the imaginary mode to understand its implications and investigate potential properties of alternative structures, determining whether the current configuration represents an averaging of two local minima.

### Elastic properties

Studying the mechanical properties of materials is crucial for assessing their potential applications within specific technical domains. These properties, intimately linked with the material's elastic constants, offer insights into various characteristics such as bonding nature, ductility, hardness, and brittleness. In an orthorhombic system, nine elastic constants (C_11_, C_22_, C_33_, C_44_, C_55_, C_66_, C_12_, C_13_, and C_23_) play critical roles in determining elastic behaviour. C_11_, C_22_, and C_33_ represent the material’s compression resistance with axes a, b, and c, whilst the remaining constants (C_44_, C_55_, C_66_, C_12_, C_13_, and C_23_) represent the crystal’s geometric properties. Furthermore, for an orthorhombic system to achieve mechanical stability, it must satisfy the subsequent Born–Huang criteria^51^ as indicated in Eqs. (1–7).

1 $$C_{11} > 0$$

2 $$C_{11} C_{22} > C_{12}^{2}$$

3 $$C_{11} C_{22} C_{33} + 2C_{12} C_{13} C_{23}$$

4 $$- C_{11} C_{23}^{2} - C_{22} C_{13}^{2} - C_{33} C_{12}^{2} > 0$$

5 $$C_{44} > 0$$

6 $$C_{55} > 0$$

7 $$C_{66} > 0$$

Elastic constant (C_ij_) for AgHgSZ (Z = Br, I) have been estimated and represented in the Table 4. Materials having larger elastic constants C_ij_ are more resistant to compression, making them less prone to deformation under external pressures. In contrast, materials with smaller elastic constants flex more easily. The C_11_ and C_33_ indicate “the resistance to uniform compression in the [100] and [001] directions”. Analysis with GGA-PBE functional demonstrates that the estimated elastic constant C_33_ for AgHgSBr and AgHgSI crystals exceeds C_11_, suggesting stronger bonding along the [001] directions than the [100] directions. The elastic constant C_44_ measures a material's capacity to endure shear deformation generated by tangential stress implemented to the [100] plane in the [010] direction. The estimated C_44_ value of AgHgSBr is lower than that of C_11_ and C_33_, implying that compression along any of its three primary crystallographic directions could lead to material deformation.

**Table 4:** The estimated elastic constants for orthorhombic structure AgHgSBr and AgHgSI using GGA-PBE functional.

Material	${C}_{11}$	${C}_{22}$	${C}_{33}$	${C}_{44}$	${C}_{55}$	${C}_{66}$	${C}_{12}$	${C}_{13}$	${C}_{23}$
AgHgSBr	40.83	82.48	65.23	25.21	21.88	18.55	28.61	34.14	26.46
AgHgSI	33.04	103.03	42.51	130.05	42.00	25.99	38.53	32.77	22.76

Conversely, for AgHgSI, C_44_ exceeds C_11_ and C_33_, suggesting that the material is less susceptible to shear deformation along these principal directions. To determine internal stresses, the Kleinman parameter ($\varsigma$) was calculated using Eq. (9)^52^. This measure offers understanding regarding a compound's ability to withstand stresses resulting from stretching and bending forces. The ($\varsigma$) values for AgHgSBr and AgHgSI suggest a limited impact of bond stretching/contracting in resisting externally applied stress. In AgHgSBr and AgHgSI, mechanical strength is primarily determined by contributions from bond stretching/contracting, with ($\varsigma$) values of 0.78 and 1.10.

8 $$\varsigma = \frac{{C_{11} + 8C_{12} }}{{7C_{11} + 2C_{12} }}$$

The elastic constants C_ij_ were used to calculate a variety of other elastic characteristics important for engineering applications, including as “bulk modulus (B), shear modulus (G), Young's modulus (E), and Poisson's ratio (n)”^21^, as described in Eqs. 9–12. Table 5 summarises the calculated “bulk moduli (B), shear moduli (G), and Young's moduli (E)” for AgHgSBr and AgHgSI crystals. Remarkably, the Young’s moduli (E) for both materials were notably higher than their bulk moduli (B) and shear moduli (G), indicating increased stiffness. Furthermore, several thermo-physical properties, such as “Young’s modulus (E) and lattice thermal conductivity (κ_L_)”, are interrelated, as expressed by κ_L_ ~ E^53^. The shear moduli (G) for AgHgSBr and AgHgSI were found to be lower than their bulk moduli (B), as shown in Table 5, indicating that shear deformation has the greatest effect on the mechanical strength of the materials examined here.

9 $$B = \frac{{B_{v} + B_{R} }}{2}$$

10 $$G = \frac{{G_{v} + G_{R} }}{2}$$

11 $$E = \frac{9GB}{{3B + G}}$$

12 $$n = \frac{3B - 2G}{{6B + 2G}}$$

**Table 5:** The calculated Bulk modulus (GPa), Young’s modulus (GPa), Shear modulus (GPa), Poisson’s ratio, and Pugh’s ratio of AgHgSBr and AgHgSI.

Material	${B}_{v}$	${B}_{R}$	$B$	${E}_{v}$	${E}_{R}$	$E$	${G}_{v}$	${G}_{R}$	$G$	${n}_{v}$	${n}_{R}$	$n$	$B/G.$
AgHgSBr	40.77	37.66	39.21	51.01	42.49	46.75	19.75	16.19	17.97	0.29	0.31	0.30	2.18
AgHgSI	40.74	32.31	36.52	99.06	12.71	55.88	45.24	4.43	24.83	0.09	0.43	0.22	1.47

To distinguish between a material's brittle and ductile qualities, one can utilise Poisson's ratio (n) and Pugh's ratio (B/G). A (B/G) ratio greater than 1.75 implies ductility, whereas values less than 1.75 show brittleness. Pure covalent materials typically have a Poisson's ratio of 0.1–0.25, whereas ionic materials have a range of 0.25 to 0.5. As a result, AgHgSBr has ionic properties and ductility, whereas AgHgSI is covalent and brittle.

Furthermore, we conducted an examination of elastic anisotropy, a significant property influencing microcrack formation. For example, Eqs. (13–15) outline the shear anisotropy ratio for the (100), (010), (001) directions. Table 6 summarises the estimated shear anisotropic factors for AgHgSBr and AgHgSI using GGA-PBE. The shear anisotropic ratio values for AgHgSBr, labelled as A_010_, are less than one, but A_100_ and A_001_ are more than one, demonstrating the anisotropy of the AgHgSBr structure. In contrast, for AgHgSI, A_100_, A_010_, and A_001_ are all above one, suggesting the anisotropic nature of the AgHgSI structure. Moreover, the “elastic anisotropy of orthorhombic structures originates from the combination of shear anisotropy and the anisotropy of the uniform bulk modulus”.

13 $$A_{100} = \frac{{4C_{44} }}{{C_{11} + C_{33} - 2C_{13} }}$$

14 $$A_{010} = \frac{{4C_{55} }}{{C_{22} + C_{33} - 2C_{23} }}$$

15 $$A_{001} = \frac{{4C_{66} }}{{C_{11} + C_{22} - 2C_{12} }}$$

**Table 6:** Elucidated shear anisotropy factors (A_100_, A_010_, A_001_) and percent (A_B_, A_G_) for AgHgSBr and AgHgSI.

Material	A_100_	A_010_	A_001_	A_B_	A_G_
AgHgSBr	2.66	0.92	1.12	0.03	0.09
AgHgSI	51.96	1.67	1.76	0.11	0.82

The variations in bulk modulus with respect to the b-axis along the a- and c-axes can be expressed using Eqs. (16 and 17). In this case, the B and G from the Voigt approximation are represented by B_V_ and G_V_, respectively, and the B and G from the Reuss approximation by B_R_ and G_R_.

16 $$A_{B} = \frac{{B_{V} - B_{R} }}{{B_{V } + B_{R} }}$$

17 $$A_{G} = \frac{{G_{V} - G_{R} }}{{G_{V } + G_{R} }}$$

When A_B_ = A_G_ = 0 (elastically isotropic), and when A_B_ = A_G_ = 1 (elastic anisotropy). The computed values of A_B_ and A_G_ for AgHgSBr and AgHgSI, as depicted in Table 6, are observed to exceed 0, signifying the anisotropic nature of both AgHgSBr and AgHgSI.

### Optical properties

**Figure 8:**
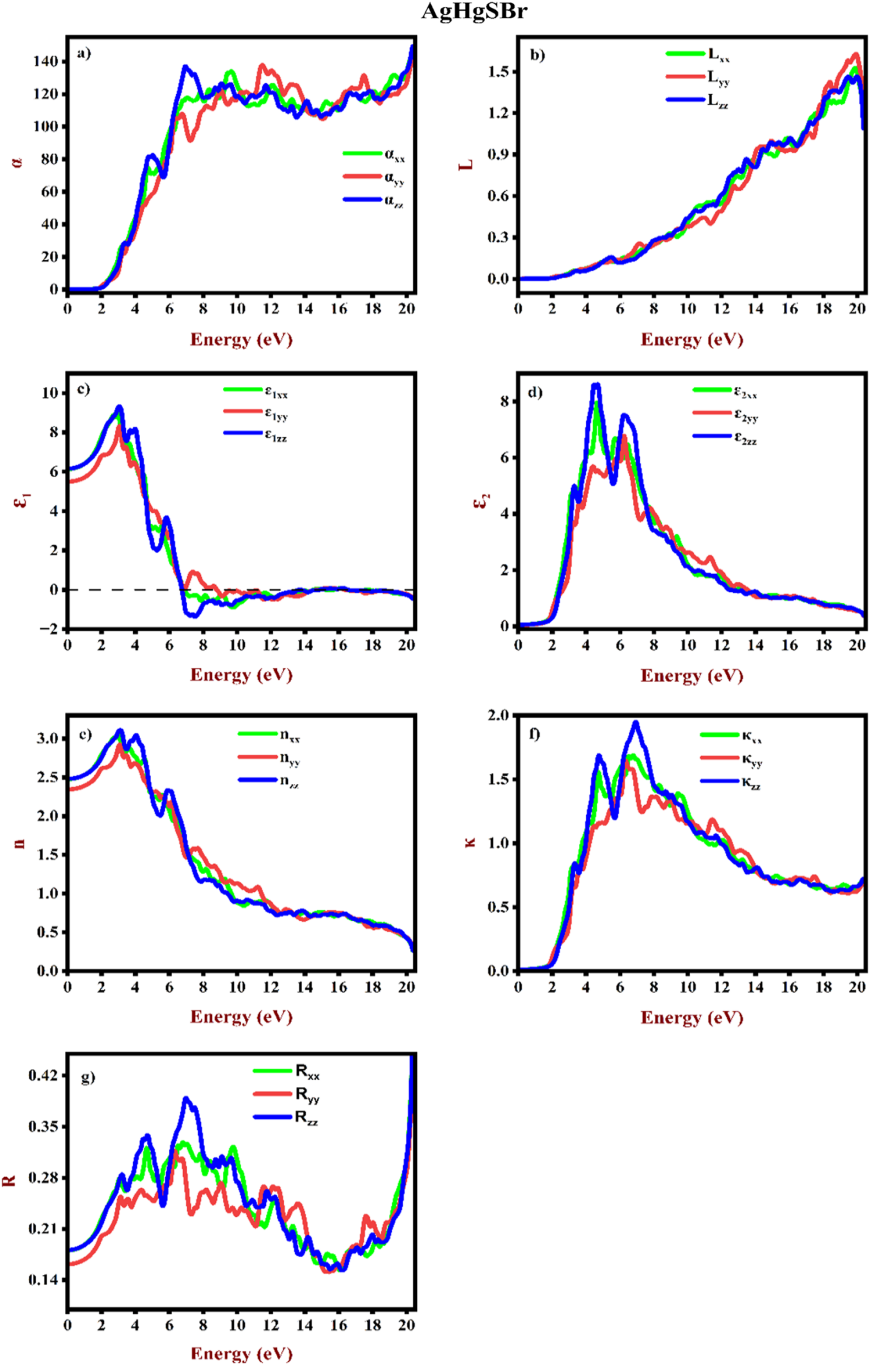
Elucidated optical properties (**a**) Absorption coefficient, (**b**) Energy loss, (**c**) and (**d**) real and imaginary dielectric constants, (**e**) Refractive index, (**f**) Extinction coefficient, and (**g**) Reflectivity for AgHgSBr.

**Figure 9:**
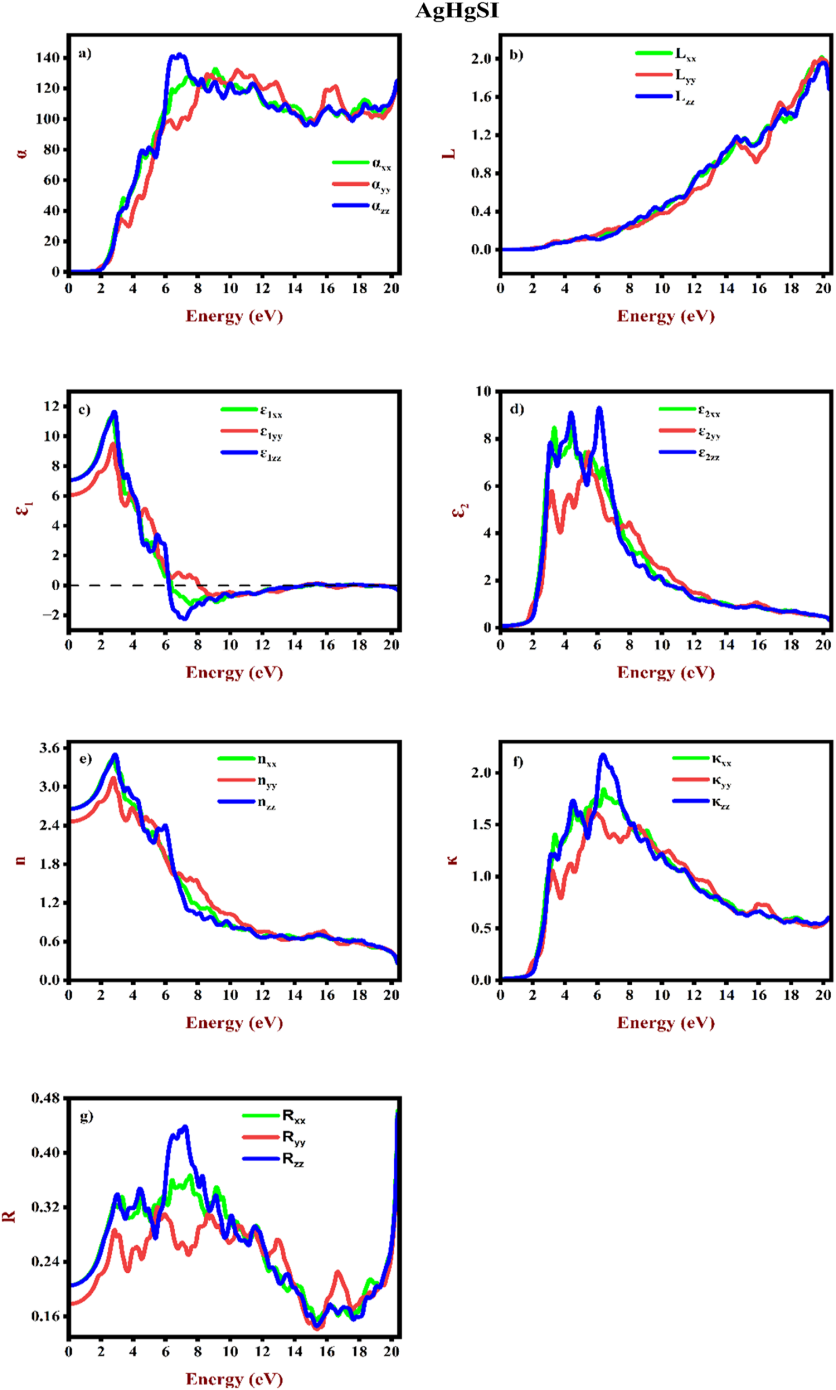
Elucidated optical properties (**a**) Absorption coefficient, (**b**) Energy loss, (**c**) and (**d**) real and imaginary dielectric constants, (**e**) Refractive index, (**f**) Extinction coefficient, and (**g**) Reflectivity for AgHgSI.

The optical properties of Mercury Sulphohalides AgHgSZ (Z = Br, I) were examined. These properties are contingent upon the interaction between electrons and photons, which governs the transition from the VB to CB. Moreover, the interband transition within semiconductor materials holds considerable importance for applications in optoelectronics^54^. Furthermore, the optical properties for AgHgSBr and AgHgSI are computed as follows: ɛ(ω) = ɛ_1_(ω) + iɛ_2_(ω), where ɛ_1_(ω) represents the real component and ɛ_2_(ω) represents the imaginary component of the dielectric function. Several optical parameters including the refractive index n(ω), absorption coefficient α(ω), reflectivity R(ω), extinction coefficient κ(ω) and energy loss L(ω) are determined as function of energy within the range 0–20 eV. These parameters are depicted in Figs. 8a–g and 9a–g. All the three polarization directions (xx, yy, zz) are taken into account. The calculation of ɛ_1_(ω) relies on the polarization of light intensity using Kramer’s-Kronig relationship.

A material’s absorption coefficient α(ω) reveals its capacity to absorb light at a certain wavelength. Figures 8a and 9a shows the computed energy-absorption relationships for AgHgSBr and AgHgSI, respectively. As energy exceeds the threshold, the absorption coefficient, α(ω), rises. The major absorption peaks for AgHgSBr are roughly 9.70, 11.52, and 6.78 eV, whereas the important peaks for AgHgSI are around 9.02, 10.40, and 6.87 eV, respectively. These materials absorb a lot of UV light, suggesting that they are not very transparent. Based on the absorption coefficient study, the examined compounds show potential for photovoltaic applications.

Figures 8b and 9b illustrate the computed electron energy loss L(ω) for the examined mercury sulphohalides complexes. Plasmon oscillation energy is predicted to occur when L(ω) reaches its peak, which corresponds to the point when the real component hits zero. Peak values represent the energy of plasmon oscillations and transitions between bands. The most notable peak in the L(ω) spectra of AgHgSBr and AgHgSI corresponds to the characteristic plasmon resonance, positioned at 19.95 eV.

The understanding of a material's electronic polarizability can be attained through the real dielectric function, as shown in Figs. 8c and 9c. The static dielectric constants ɛ_1_(0) along the three crystallographic directions are determined to be 6.14 (xx), 5.50 (yy), and 6.16 (zz) for AgHgSBr, and 7.07 (xx), 6.05 (yy), and 7.06 (zz) for AgHgSI, respectively. The average values of the zero-frequency dielectric constants ɛ_1_(0) for AgHgSBr and AgHgSI are 5.93 and 6.72, respectively. Nevertheless, there is no experimental polarized zero-frequency dielectric constant available for comparison. These findings distinctly indicate anisotropy in the optical characteristics of orthorhombic AgHgSZ (Z = Br, I). The ratio $\frac{{\upvarepsilon }_{1}^{yy} (0)}{{\upvarepsilon }_{1}^{zz} (0)}$ is calculated to be 0.892 for AgHgSBr and 0.856 for AgHgSI to assess the degree of anisotropy. Commencing from the zero-frequency limit, in AgHgSBr, they steadily increase until they reach peak values of 8.97 at 2.97 eV (xx), 8.32 at 3.06 eV (yy), and 9.33 at 3.03 eV (zz), respectively. Similarly, in AgHgSI, they begin to ascend and peak at maximum values of 11.26 at 2.73 eV (xx), 9.50 at 2.73 eV (yy), and 11.66 at 2.81 eV (zz). These peaks represent the distribution of light intensity polarisation across different frequencies inside the materials. The polarisation curve reaches its lowest point when there is a little shift in the resonance frequency value. The materials under investigation exhibits semiconductor-like response to incident light. In the case of AgHgSBr, the energy values are 6.87 eV to 20.36 for xx, 8.80 eV to 20.36 eV for yy, and 6.76 eV to 20.36 eV for zz indicates negative values. Similarly, for AgHgSI the energy values are 6.38 eV to 20.36 eV for xx, 8.06 eV to 20.36 eV for yy, 6.21 eV to 20.36 eV exhibits negative values.

Both of the halides (AgHgSBr and AgHgSI) under study undergo a transition where their real dielectric values become negative as the energy level rises over 6 eV. The electromagnetic waves are hindered in their propagation by this negative dispersion feature. The material's characteristics shift from dielectric to metallic as a result. Furthermore, as can be shown in Figs. 8g and 9g, reflectivity must be maximum in the negative regions of epsilon (real) due to the inverse relationship between reflectivity and real epsilon. The energy-wise absorption sections of ɛ_2_(ω) are depicted in Figs. 8d and 9d. There are direct transitions between bands indicated by peaks in the imaginary components of ɛ_2_(ω). AgHgSBr and AgHgSI threshold energies are determined to be 1.833 and 1.832 eV, respectively. Both of ɛ_2_(ω)'s components improve beyond these points. Figures 8e and 9e illustrate a gradual rise in the parallel components of the static refractive index n(0), for each of the materials under investigation as energy escalates, ultimately reaching peak values. However, when energy levels climb, both components of the refractive index decline significantly, occasionally going below unity at energies of 9 eV and 7 eV for AgHgSBr and AgHgSI, respectively. The calculated values for both components of the static refractive index, n(0), which are 2.47 (xx), 2.35 (yy), and 2.48 (zz) for AgHgSBr and 2.65 (xx), 2.46 (yy), and 2.65 (zz) for AgHgSI. Both compounds' plots show a minor anisotropy in the xx, yy, and zz directions.

The imaginary component of refraction, κ(ω), also known as the extinction coefficient, represents the loss in light energy as it travels through materials. Figures 8f and 9f show determined values for κ(ω). These plots closely resemble those of ε_2_(ω), ensuring the precision of calculations. Whether measured through dielectric constants or the refraction process, the findings yield identical results in terms of the decay of light energy. κ(ω) are depicted in Figs. 8f and 9f. These plots closely resemble those of ε_2_(ω), ensuring the precision of calculations. Whether measured through dielectric constants or the refraction process, the findings yield identical results in terms of the decay of light energy. The estimated reflectivity R(ω) spectra, shown in Figs. 8g and 9g shows that the compounds under investigation found near-UV reflectivity values ranging from around 30% to 40%. Reflectivity increases with energy levels, peaking at 50% at 20.36 eV. This discovery shows that these materials might be good protective coatings in the energy spectrum with the maximum reflecting characteristics. AgHgSBr has zero-frequency reflectivity values of 18%, 16%, and 18% at xx, yy, and zz. Similarly, AgHgSI possesses zero-frequency reflectivity values of 20%, 17%, and 20% for xx, yy, and zz, respectively.

### Transport properties

**Figure 10:**
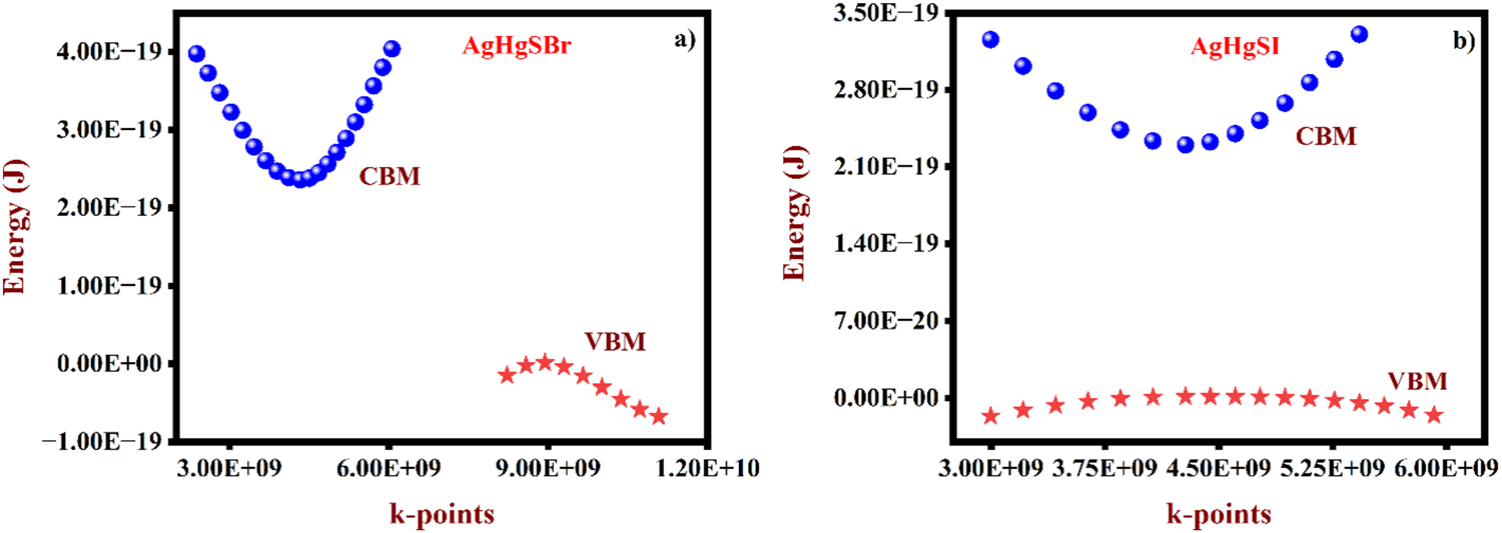
The estimated VBM and CBM for (**a**) AgHgSBr and (**b**) AgHgSI.

Band structures made simpler by comparing their effective mass to the behaviour of a free element of that mass. We study the effective mass of AgHgSZ (Z = Br, I) compounds to examine the influence of substitution on conductivity, using the theory of effective mass as a direct tool for understanding the electrical characteristics of materials. In order to calculate the effective band mass, one must look at the band's curvature at point Γ, which is the extreme point in k-space. To put it simply, parabolic curves given in Eq. (18) match certain band mass components, including the valence band maximum (VBM) and conduction band minimum (CBM) seen in Fig. 10a,b:

18 $$\frac{1}{{{\text{m}}_{{{\text{eff}}}} }} = \frac{1}{{\hbar^{2} }}\frac{{\partial^{2} {\text{E}}}}{{\partial {\text{k}}^{2} }}$$

The Table 7 shows effective masses (m_eff_) that in AgHgSBr and AgHgSI, electrons have lower effective masses than holes, implying that electrons may have more mobility. The band structures shown in Fig. 4 for AgHgSBr and AgHgSI indicate a p-type semiconductor, implying that holes are the favoured charge carriers. As a result, holes have a higher effective mass than electrons do. Equation (19) makes it easier to calculate exciton binding energy, with values for AgHgSBr and AgHgSI estimated at 36.3, 45.2, and 36.1 meV, as well as 24.2, 32.9, and 24.3 meV across the x, y, and z directions of the ɛ_1_(0). Using Eq. (20)^55^, the exciton Bohr radius may be computed, obtaining values for AgHgSBr and AgHgSI of 32.24 Å, 28.87 Å, and 32.34 Å, 42.05 Å, 36.03 Å, and 41.99 Å, in the x, y, and z directions. Interestingly, AgHgSBr and AgHgSI indicates a lower exciton binding energy throughout, with its a* value exceeding the lattice parameters exhibits as a weak exciton of the Mott-Wannier type.

19 $$E_{b} = { }13.6\frac{{m_{\mu } }}{{m_{0} }}\frac{1}{{\varepsilon_{1} \left( 0 \right)^{2} }}$$

20 $$a^{*} { } = { }\varepsilon_{1} \left( 0 \right)\frac{{m_{0} }}{{m_{\mu } }}a_{0}$$

**Table 7:** The estimated effective masses for AgHgSBr and AgHgSI compounds.

Material	Electron	Hole
AgHgSBr	0.1210 ${m}_{e}$	0.6051 ${m}_{e}$
AgHgSI	0.1008 ${m}_{e}$	0.7563 ${m}_{e}$

The exploration of the electronic behavior and optical properties suggests that AgHgSBr and AgHgSI hold potential for photovoltaic applications. Harnessing waste heat to generate electrical energy and prevent electronic devices from overheating by converting this thermal energy into electricity represents an emerging domain within renewable energy. The effectiveness of AgHgSBr and AgHgSI are evaluated using figure of merit (ZT), calculated as (S^2^σ/κ). Therefore, achieving high ZT values relies on maximizing the potential gradient between the metal contacts, which is computed by the Seebeck coefficient (S) and electrical conductivity (σ) at room temperature. The thermal conductivity, comprising electronic (κ_e_) and phononic contributions (κ_L_), is also addressed in this context.

**Figure 11:**
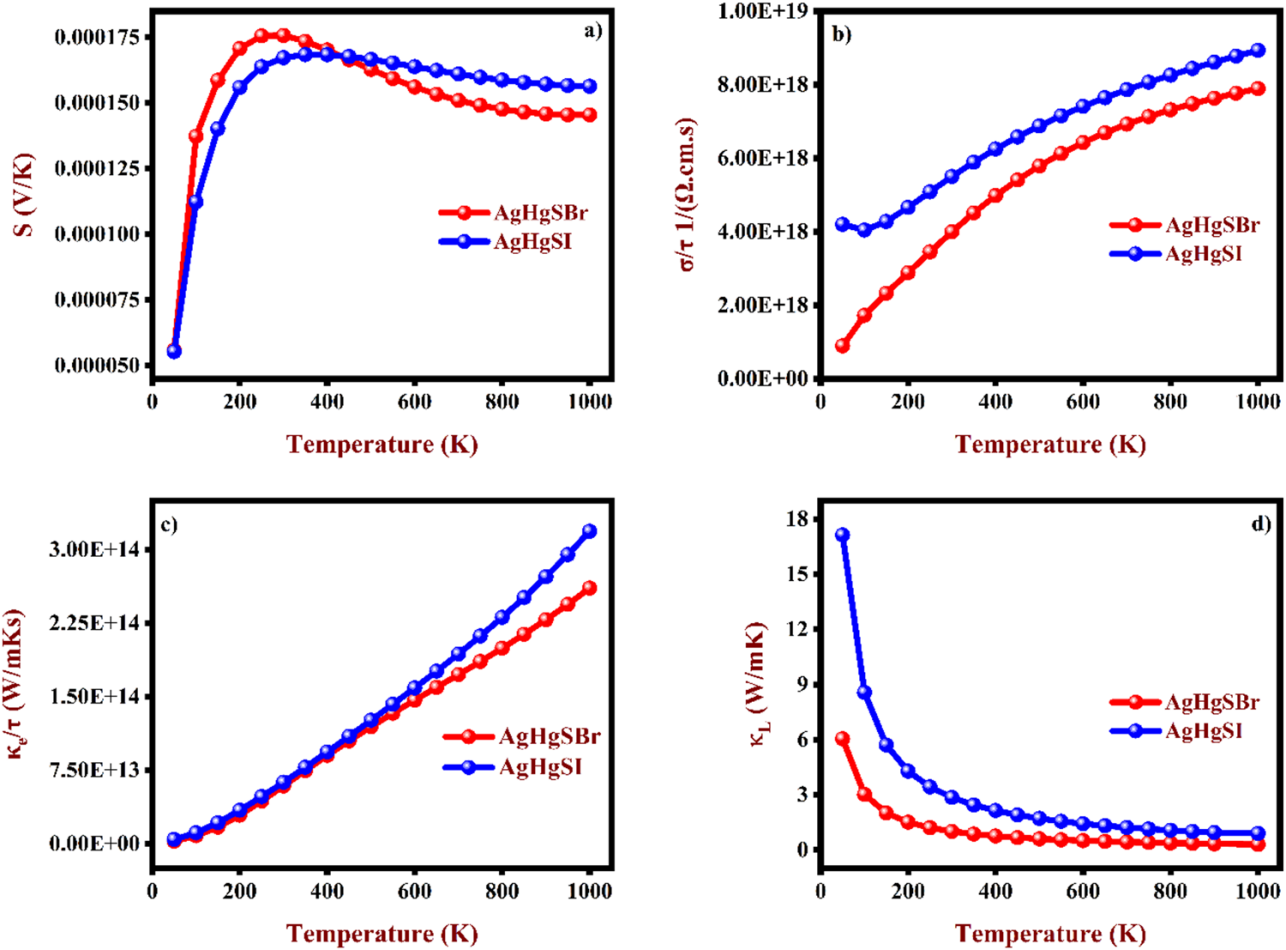
The estimated thermoelectric properties (**a**) Seebeck coefficient, (**b**) Electrical conductivity, (**c**) Electronic thermal conductivity, and (**d**) Lattice thermal conductivity for AgHgSBr and AgHgSI.

**Figure 12:**
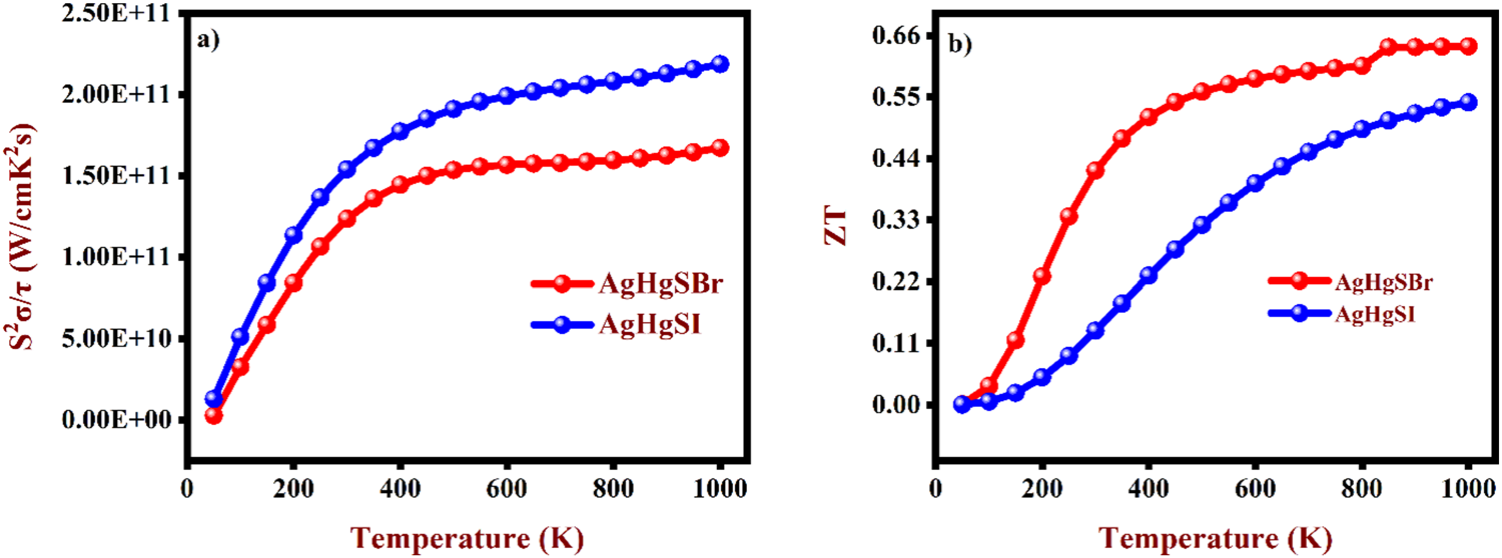
The estimated (**a**) Power factor, and (**b**) ZT for AgHgSBr and AgHgSI.

Thermoelectric evaluations for AgHgSBr and AgHgSI are done using Boltzmann transport theory, which is based on frequency band dispersion. This study examines σ, κ_e_, and κ_L_ in relation to relaxation time (t = 10^–14^ s), which is factored into the computation method. The thermoelectric parameters are illustrated in Figs. 11a–d, 12a,b, thoroughly discussed below. In this research, the investigators examined the fluctuations in thermoelectric (TE) properties across a temperature range spanning from 50 to 1000 K.

The Seebeck coefficient (S), also known as thermopower, is a thermoelectric material's capacity to create an electric current in reaction to a change in temperature. This factor is crucial in assessing the effectiveness of thermocouples and other thermoelectric apparatus. In Fig. 11a, the Seebeck coefficient for AgHgSZ (Z = Br, I) compounds predominantly carry holes as charge carriers. This implies a surplus of positively charged carriers compared to electrons within these materials. When electrons went from VB to CB, holes are created. The Seebeck coefficient's positive value denotes an excess of holes relative to electrons since it is proportional to the number of charge carriers. Because of their weaker atomic affinity, holes are more mobile than electrons, which increases how useful they are for producing energy. This positive thermopower (S) raises the prospect of thermoelectric applications, wherein devices that generate power from temperature changes employ the Seebeck effect. Higher S denote increased thermoelectric device efficiency. Therefore, the positive Seebeck coefficient observed in AgHgSZ (Z = Br, I) compounds underscores their promise for such applications. Additionally, the Fig. 11a illustrates substantial variations in the Seebeck coefficient of AgHgSBr and AgHgSI with rising temperature. At ambient temperature, AgHgSBr and AgHgSI exhibit Seebeck coefficients of 176 μV/K and 167 μV/K, respectively. However, at a significantly elevated temperature of 1000 K, the S values decrease to 145 μV/K for AgHgSBr and 156 μV/K for AgHgSI. The Seebeck coefficient of AgHgSBr demonstrates a gradual rise up to 300 K, followed by a slow decline until reaching 1000 K. Similarly, for AgHgSI, the S exhibits an increase up to 350 K, with a subsequent gradual decrease observed up to 1000 K.

The electrical conductivity (σ) of thermoelectric materials pertains to their ability to transport electrical charge. Typically, semiconductor materials can have their electrical conductivity adjusted through a process known as doping, which entails introducing impurities to modify their electrical properties. The level of electrical conductivity holds significance in thermoelectric materials as it directly impacts the efficiency of the thermoelectric phenomenon. In our investigation, the electrical conductivity of AgHgSZ (Z = Br, I) compounds under scrutiny is illustrated in Fig. 11b. The graph depicts a linear augmentation in electrical conductivity for both materials as temperature increases, attributed to the escalating clusters of charge carriers. Notably, AgHgSI demonstrates higher electrical conductivity compared to AgHgSBr, a characteristic contrasting with the temperature-dependent alterations observed in thermopower a common trait of semiconducting materials. At 1000 K, both materials exhibit a maximum electrical conductivity nearing 7.890 × 10^–18^ 1/(Ω.cm.s) and 8.940 × 10^–18^ 1/(Ω.cm.s), respectively.

Thermal conductivity refers to a material’s capacity to transmit heat by measuring the rate of heat transfer across a given area per unit temperature differential. Watts per metre Kelvin (W/mK) is a popular unit of measurement. Elevated thermal conductivity indicates enhanced efficiency in heat propagation. Consequently, materials exhibiting high thermal conductivity find utility in applications necessitating rapid heat dissipation, such as heat sinks. Conversely, materials characterized by low thermal conductivity serve as effective insulators, impeding the transfer of heat between distinct locations. The lattice vibrations associated with atomic mobility in semiconductor materials increase as temperatures rise. Furthermore, the movement of heat energy by convection from locations with greater to lower potential accentuates this impact. The interplay between phonons and electrons estimates semiconductor thermal conductivity. The temperature dependent electronic thermal conductivity (κ_e_) as shown in Fig. 11c, demonstrating that the κ_e_ of AgHgSZ (Z = Br, I) materials escalates with increasing temperature. The value κ_e_ at room temperature for AgHgSBr and AgHgSI are 5.91 × 10^–13^ (W/mKs) and 6.28 × 10^–13^ (W/mKs).

The lattice thermal conductivity stands as a fundamental property of solids. In this study, we have examined the fluctuation in the lattice thermal conductivity (κ_L_) of our compounds across varying temperatures as shown in Fig. 11d, employing the Slack model^49^ in Eq. (21).

21 $$\kappa_{l} = A\frac{{M_{a} \theta^{3} \delta }}{{\gamma^{2} Tn^{2/3} }}$$

In the aforementioned equation, the symbols “A, M_a_, θ, δ, γ, and n stand for respective physical parameters: average atomic mass, Debye temperature, cube root of the average atomic volume, Grüneisen parameter, and the number of atoms in a primitive unit cell”^21^. The determination of the physical quantity A involves a calculation based on the following relationship in Eq. (22).

22 $$A = \frac{{2.43 \times 10^{ - 8} }}{{1 - \frac{0.514}{\gamma } + \frac{0.228}{{\gamma^{2} }}}}$$

For AgHgSBr and AgHgSI, we determined the Grüneisen parameter (γ) and Debye temperature (θ) using the WIEN2k-interfaced IRelast package^45^. For AgHgSBr, we determined θ to be 184.28 K, accompanied by a γ value of 1.78. Similarly, for AgHgSI, we calculated θ as 206.71 K, with a corresponding γ of 1.37. As temperature rises, the lattice contribution to thermal conductivity (κ_L_) decreases in both compounds. Specifically, at 300 K, κ_L_ is approximately 1.01 (W/mK) for AgHgSBr, declining to 0.30 (W/mK) at 1000 K. Similarly, for AgHgSBr, κ_L_ is around 2.86 (W/mK) at 300 K, dropping to 0.85 (W/mK) at 1000 K. Presently, there are no experimental values available for comparison. When compared to other quaternary coinage compounds that have low lattice thermal conductivity, such as the newly found CuHgSeBr (with a value of 0.69 W/mK at 300 K), it appears to be lower than AgHgSBr and AgHgSI. The increase in electronic thermal conductivity is caused by the formation of electron–hole pairs as a result of heat, resulting in a decrease in the mean free path of phonons and a considerable drop in κ_L_.

Furthermore, Fig. 12a indicates the computed relationship of the power factor (PF) with temperature, it is calculated as PF = S^2^σ. As seen in Fig. 12a, the PF increases significantly as temperature rises, culminating at high temperatures. This tendency results from the simultaneous increase in carrier concentration and growing temperature. Peak power factor is associated with a lower S and higher σ. This behaviour highlights how important it is to strike a balance between these two elements in order to improve thermoelectric material power production efficiency.

Without a doubt, the most crucial metric for determining the thermoelectric characteristics of materials is the ZT. Using formula ZT = S^2^σ/κ. Figure 12b shows how the figure of merit (ZT) fluctuates in response to temperature. Notably, it is discernible that both the AgHgSBr and AgHgSI compounds exhibit an upward trend in ZT with increasing temperature. This observed augmentation in ZT with temperature for both AgHgSBr and AgHgSI suggests an enhancement in their thermoelectric efficiency as temperature escalates. The recorded ZT of 0.41 and 0.13 at room temperature for AgHgSBr and AgHgSI, respectively, underscore the significance of these studied materials. Furthermore, juxtaposing with CuHgSeBr^22^, the ZT values are notably higher for AgHgSBr and comparatively lower for AgHgSI. Based on this ZT analysis, our investigated compounds demonstrate suitability for thermoelectric applications, particularly at elevated temperatures.

## Conclusion

In this comprehensive investigation, we delve deeply into the optical, electronic, elastic, and thermoelectric properties of AgHgSBr and AgHgSI materials through meticulous density functional theory (DFT) computations. Employing the GGA-PBE method, we establish band gap values of 1.833 eV for AgHgSBr and 1.832 eV for AgHgSI. Furthermore, we evaluate the dynamic and structural stability of these compounds. Our analysis of their elastic properties confirms the mechanical resilience and ductility of AgHgSBr, in stark contrast to the brittle nature of AgHgSI, indicative of a prevalent combination of ionic and covalent bonding traits. Additionally, considering their anticipated band gap, high refractive index, and significant absorption coefficient, particularly in the UV spectrum, our findings unveil compelling prospects for photovoltaic applications. Moreover, we meticulously investigate the thermoelectric characteristics of AgHgSZ (Z = Br, I) using the semi-classical BoltzTrap method. Through a detailed examination of the band structure, we derive crucial parameters such as effective mass and exciton binding energy. Depending on the exciton Bohr radius, our results suggest the presence of a Mott-Wannier type excitons. Notably, both materials show considerable potential as thermoelectric devices, as evidenced by power factor and ZT measurements. This extensive study significantly advances the field of materials science by enriching our understanding and application of AgHgSBr and AgHgSI materials in photovoltaic and thermoelectric domains. Moreover, our theoretical findings provide a foundation for experimental researchers to further explore the thermoelectric and optical properties of these materials.

## Data Availability

The datasets used and/or analysed during the current study available from the corresponding author on reasonable request.
